# Deep Convolutional Neural Network-Assisted Feature Extraction for Diagnostic Discrimination and Feature Visualization in Pancreatic Ductal Adenocarcinoma (PDAC) versus Autoimmune Pancreatitis (AIP)

**DOI:** 10.3390/jcm9124013

**Published:** 2020-12-11

**Authors:** Sebastian Ziegelmayer, Georgios Kaissis, Felix Harder, Friederike Jungmann, Tamara Müller, Marcus Makowski, Rickmer Braren

**Affiliations:** 1 Department of Diagnostic and Interventional Radiology, School of Medicine, Technical University of Munich, 81675 Munich, Germany; ga89rog@mytum.de (S.Z.); g.kaissis@tum.de (G.K.); felix.harder@tum.de (F.H.); f.jungmann@t-online.de (F.J.); tamara.mueller@tum.de (T.M.); marcus.makowski@tum.de (M.M.); 2 Department of Computing, Faculty of Engineering, Technology and Medicine, Imperial College of Science, London SW7 2BU, UK; 3 German Cancer Consortium, Partner Site Technical University of Munich, D-69120 Heidelberg, Germany

**Keywords:** deep learning, radiomics, pancreatic cancer, autoimmune pancreatitis

## Abstract

The differentiation of autoimmune pancreatitis (AIP) and pancreatic ductal adenocarcinoma (PDAC) poses a relevant diagnostic challenge and can lead to misdiagnosis and consequently poor patient outcome. Recent studies have shown that radiomics-based models can achieve high sensitivity and specificity in predicting both entities. However, radiomic features can only capture low level representations of the input image. In contrast, convolutional neural networks (CNNs) can learn and extract more complex representations which have been used for image classification to great success. In our retrospective observational study, we performed a deep learning-based feature extraction using CT-scans of both entities and compared the predictive value against traditional radiomic features. In total, 86 patients, 44 with AIP and 42 with PDACs, were analyzed. Whole pancreas segmentation was automatically performed on CT-scans during the portal venous phase. The segmentation masks were manually checked and corrected if necessary. In total, 1411 radiomic features were extracted using PyRadiomics and 256 features (deep features) were extracted using an intermediate layer of a convolutional neural network (CNN). After feature selection and normalization, an extremely randomized trees algorithm was trained and tested using a two-fold shuffle-split cross-validation with a test sample of 20% (*n* = 18) to discriminate between AIP or PDAC. Feature maps were plotted and visual difference was noted. The machine learning (ML) model achieved a sensitivity, specificity, and ROC-AUC of 0.89 ± 0.11, 0.83 ± 0.06, and 0.90 ± 0.02 for the deep features and 0.72 ± 0.11, 0.78 ± 0.06, and 0.80 ± 0.01 for the radiomic features. Visualization of feature maps indicated different activation patterns for AIP and PDAC. We successfully trained a machine learning model using deep feature extraction from CT-images to differentiate between AIP and PDAC. In comparison to traditional radiomic features, deep features achieved a higher sensitivity, specificity, and ROC-AUC. Visualization of deep features could further improve the diagnostic accuracy of non-invasive differentiation of AIP and PDAC.

## 1. Introduction

Autoimmune pancreatitis (AIP) is a rare inflammatory disease that can be classified into two subtypes. AIP type 1 is associated with increased levels of IgG-4 and typically shows other organ involvement; in contrast, AIP type 2 commonly affects only the pancreas. While different diagnostic guidelines were established [1,2,3], the accuracy of these diagnostic scoring systems remains unsatisfactory, with some patients not matching any of the common scoring systems [4,5]. Moreover, different studies show that cases of AIP are at risk for being misdiagnosed as pancreatic ductal adenocarcinoma (PDAC), falsely leading to pancreatic resection [6,7]. This is mainly due to the overlapping clinical and radiological features of both entities. AIP patients commonly present with painless jaundice, making malignancy of the pancreaticobiliary system a likely differential. While AIP typically responds well to corticosteroid therapy, PDACs require resection and/or chemotherapeutic treatment. Computed tomography (CT) is frequently used in the work-up of patients presenting with painless jaundice and different imaging parameters are already implemented in the above-mentioned scoring systems. While some imaging characteristics are suggestive of AIP, for example, “sausage-like” enlargement of the pancreas, AIP can also cause focal mass or abnormal enhancement, making it difficult to differentiate it from pancreatic cancer.

In recent years, radiomics, a computer-based analysis of quantitative imaging features, has been widely used to increase diagnostic accuracy and successfully predict patient outcome and therapy response. Recent studies show that a differentiation between AIP and PDAC based on radiomic features is possible [8,9,10]. Furthermore, radiomic features were successfully used to predict therapeutically relevant subtypes of PDAC, and overall and progression-free survival of PDAC patients [11,12].

Convolutional neural networks (CNNs), which have been used for image classification tasks to great success, are being increasingly applied for medical image analysis, outperforming traditional machine learning (ML) algorithms on large datasets [13,14]. However, small datasets and label uncertainty, a typical limitation seen in the medical field, can hinder the successful end-to-end training of CNNs. Alternatively, CNNs pretrained on a large dataset, for example ImageNet [15], can be used as a feature extractor. These features (deep features) provided promising results for different tasks in image analysis [16,17]. However, few studies have investigated the utility of deep features in medical imaging.

In our study, we trained a machine learning model using deep features and traditional radiomic features and compared their predictive value in the differentiation of AIP and PDAC in portal venous CT-scans.

## 2. Experimental Section

The study was designed as a retrospective cohort study. Ethics approval for patient recruitment and data processing and analysis was obtained (180/17), and the requirement for individual written consent was waived. All procedures and analyses were carried out in accordance with pertinent laws and regulations. A patient recruitment flowchart and the STROBE (The STrengthening the Reporting of OBservational studies in Epidemiology) checklist [18] are included in the Supplementary Materials. A total of 86 patients, 44 with AIP and 42 with PDAC, were found eligible. Patients without a baseline CT-scan, inadequate or incomplete imaging data, and insufficient ground truth data were excluded. AIP was either diagnosed histopathologically (*n* = 22) or based on a combination of serology, imaging data, and therapy response to corticosteroids (*n* = 22) in accordance with the international consensus diagnostic criteria (ICDC) for autoimmune pancreatitis [1]. PDACs were histopathologically diagnosed after tumor resection and the pathological parameters were noted. Based on the 7th edition of the TNM-staging for pancreatic ductal adenocarcinoma, only PDACs limited to the pancreas (tumor stage (T): 1/2) and without regional lymph node metastasis (nodal status (*n*): 0) were selected. Age at diagnosis, sex, localization, diffuse or focal involvement (if applicable), and suspected malignancy were noted for each patient. For the clinical confounders, the chi-squared test and the Students t-test were used for cross-tabulations and continuous variables, respectively. A significance level of *p* < 0.05 was chosen.

Participants were screened for eligibility based on a search of the hospital picture archiving system (PACS). A total of 86 patients with portal venous CT-scans (70 s post injection of iodinated contrast media) were obtained between the 1 November 2004 and 1 March 2020. The imaging data were exported in pseudonymized form. Pancreas segmentation was done automatically using an in-house algorithm. Each segmentation mask was controlled under radiological reporting conditions by two experienced observers (S.Z., G.K.); if necessary, segmentation masks were adapted using ITK-SNAP v. 3.8.0 (Penn Image Computing and Science Laboratory (PICSL), University of Pennsylvania, PA, USA and Scientific Computing and Imaging Institute (SCI), University of Utah, UT, USA) [19].

Radiomic feature extraction was done using the Python package PyRadiomics v 3.0 [20]. The detailed settings for the feature extraction can be found in the Supplementary Materials. In total, 1411 features were extracted from the CT-images. The following feature preprocessing steps were applied to eliminate unstable and non-informative features. Low variance features (below 0.1) were excluded. Correlation of the remaining 943 features was calculated using Spearman’s correlation coefficient; features with a correlation coefficient above or below ± 0.9 were excluded. The resulting 299 features were normalized to the (0.1) interval.

For the deep-feature extraction, a VGG19 (CNN architecture which consist of 19 layers (16 convolution layers, 3 Fully connected layer, 5 MaxPool layers, 1 SoftMax layer) [21] pretrained on ImageNet was used. For every patient, the CT-image-stack was resized and extended by two channels to match the input shape of 224 (height) × 224 (width) × 3 (channels). The Hounsfield units were rescaled to (0,255). For each CT-image, 256 feature maps of shape 56 × 56 were calculated and extracted on the layer “block3conv4”, yielding 590,080 parameters. The feature maps were summed and normalized to the (0.1) interval. The mean value was calculated for every feature map, yielding 256 features per patient. Features were preprocessed as described for the radiomic features, yielding 79 features. Feature maps were plotted, and visual differences were noted by two experienced radiologists (S.Z., G.K.).

For the prediction of AIP or PDAC, an extremely randomized trees classifier [22] was fit on the radiomic and deep features. Hyperparameter tuning was performed using grid search with a 5-fold cross-validation. The best parameters were retained (hyperparameters can be found in the Supplementary Materials. Two-fold shuffle-split cross-validation with a test sample fraction of 0.2 was performed and the average sensitivity, specificity and ROC-AUC were obtained. ROC curves of the average performance of the algorithm were plotted for both feature groups. The permutation feature importance was assessed using the feature importance function from scikit-learn [23]. All statistical and machine learning analysis was performed using Python v.3.7.9 (Python Software Foundation, DE, USA) and Keras with a TensorFlow [24] backend.

## 3. Results

In total, 86 patients (AIP: *n* = 44, PDAC: *n* = 42) were analyzed. The distribution of the clinical and histological parameters can be found in Table 1. Of the 42 resected PDACs, 14 were classified as T1 and 28 as T2 tumors. All PDACs were classified as focal lesions.

**Figure 1:**
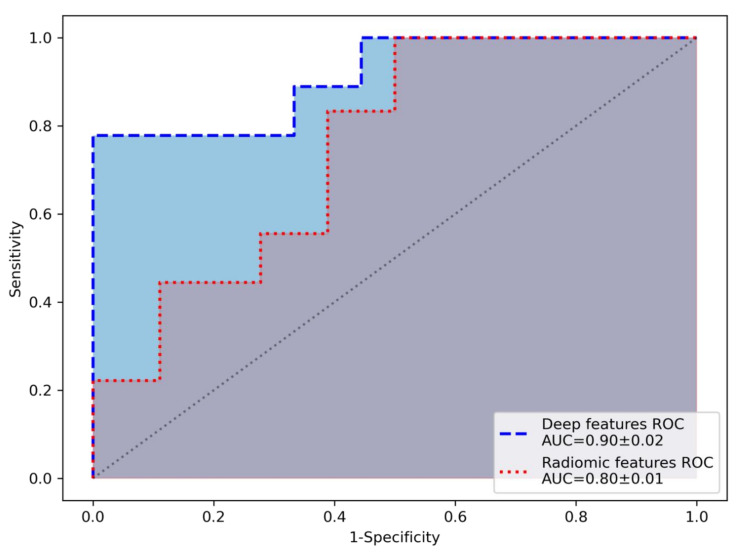
Receiver operating characteristic curve (ROC) of the performance of the ML algorithm, showing an area under the curve (AUC) of 0.9 for the deep features and 0.8 for the radiomic features.

After feature extraction and selection, the extremely randomized trees algorithm was trained on deep features (*n* = 79) and radiomic features (*n* = 299), achieving higher sensitivity (mean ± std), specificity (mean ± std) and ROC-AUC (mean ± std) over the cross-validation folds for the deep features (0.89 ± 0.11, 0.83 ± 0.06, and 0.90 ± 0.02) compared to radiomic features (0.72 ± 0.11, 0.78 ± 0.06, and 0.80 ± 0.01). ROC curves of the average performance of the machine learning algorithm for the deep features and radiomic features are shown in Figure 1.

**Figure 2:**
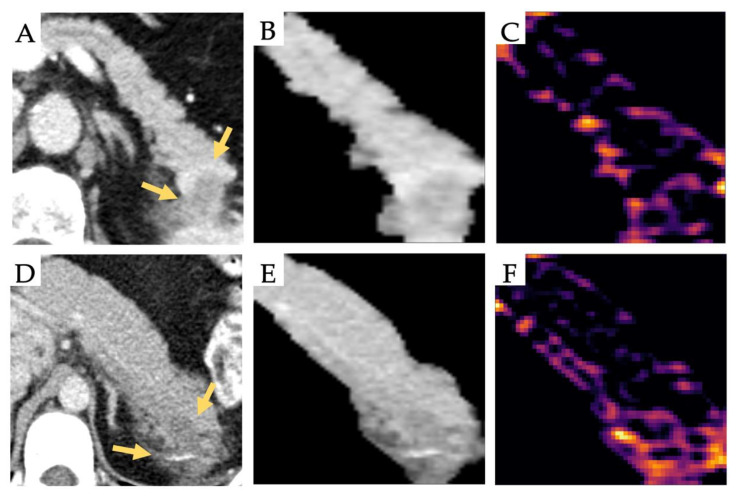
Representative CT-images (Yellow arrows pointing to the lesion) of a pancreatic ductal adenocarcinoma (PDAC) (**A**) and an autoimmune pancreatitis (AIP) (**D**) and their corresponding segmentation image (**B**,**E**) and feature map (**C**,**F**). For the AIP lesion, the activation of the feature map appears to focus on the internal structure; in contrast, activation is noted at the transition zone between the lesion and normal tissue for the PDAC.

Age as a clinical feature was significantly different between groups (*p* = 0.001). The analysis was repeated for the deep features and the radiomic features with age as an additional feature. No increase in sensitivity, specificity, and ROC-AUC was observed. Feature permutation importance was computed. No feature reached an importance above 0.056 for the deep features and 0.022 for the radiomic features. The feature importance of age was 0.00 for both the deep features and radiomic features, indicating that age did not contribute to the accuracy of the prediction. The 256 feature maps were plotted, and visual differences were noted. A visualization of a subset (*n* = 64) of the feature maps for exemplary patients can be found in the Supplementary Materials. Figure 2 shows CT-images of exemplary patients and the visualization of a representative feature map, indicating different activation patterns. In the displayed feature map, the PDAC lesion shows the highest activation in the transition zone between normal and tumor tissue, whereas the primary lesion shows no activation. In contrast, the AIP lesion shows activation in the inner structure of the lesion and low activation in the transition zone. This indicates that the deep neural network learns features discriminative between AIP and PDAC, owing to lesion architecture manifesting as imaging features.

## 4. Discussion

Due to overlapping clinical and imaging characteristics of AIP and PDAC, safely ruling out malignancy in AIP patients is often difficult. Misdiagnosis can dramatically worsen the outcome of both entities. Therefore, further diagnostic tools are needed that increase diagnostic accuracy.

In this study, we compared the predictive value of deep features and radiomic features in the differentiation of AIP and PDAC in portal venous CT-scans using a machine learning algorithm. For our cohort, the prediction based on the deep features achieved a higher sensitivity, specificity, and ROC-AUC compared to handcrafted radiomic features.

Radiomic feature analyses have been used for different tasks in a variety of radiological datasets, achieving promising results, including the differentiation between AIP and PDAC [8,9,10]. In the cited publications, the authors were able to predict AIP and PDAC on CT/PET-CT images with high sensitivity and specificity by combining traditional radiomics feature extraction and selection with a ML model. However, there are certain drawbacks inherent to the methodology of radiomics studies. Radiomic features are based on predefined functions and, therefore, are highly dependent on the defined region of interest (ROI), which requires profound domain knowledge and can be time consuming depending on the sample size. Dependency on bin number and ROI size further complicates a standardization [25,26]. Furthermore, different phantom studies have shown that even under experimental conditions, radiomic features can be unstable [27,28]. In combination with the loss of information, due to the spatial and intensity discretization and the limited complexity of the features, radiomic features may only capture a rudimentary representation of the input image. In contrast, CNNs combine low- and high-level features, which results in a more abstract and complex representation of the input image. CNN-based features are learned specifically to improve the performance on the task given and are therefore more versatile in use, which also allows a transfer of features that have been trained on a similar task. In our study, we used a VGG19 pretrained for image classification on the ImageNet dataset. Several studies have shown that using deep features of a pretrained CNN combined with a machine learning classifier can achieve high sensitivity and specificity on different imaging tasks [29,30] and permit generalization to various other tasks [31].

A major challenge in the successful translation of radiomics and deep learning into clinical practice is the lack of visualization and interpretability. In contrast to radiomic features, the feature maps of CNNs can be plotted for visual interpretation by an observer. In our study, we visualized the 256 feature maps and noted that individual feature maps enable a visual differentiation of both entities. The feature map presented in Figure 2 indicates activation in the internal structure of the AIP lesion and a more peripheral located activation for the PDAC, which is in line with a recent study on pancreatic findings in endoscopic ultrasound images in which high-scoring CNN activation was centrally located in the pancreas parenchyma for AIP [32]. The influence of these feature maps on radiological decision-making needs to be investigated in further studies. A recent study has shown that deep features can be replaced with semantic features defined by a radiologist, indicating the discriminatory ability of deep features and their potential to be visually interpretable [33].

The following limitations of our study have to be addressed. The retrospective nature and small patient cohort limit the generalizability of the results and would require multi-center prospective studies to validate the importance of deep features in the differentiation of AIP and PDAC. Consequently, a transfer learning approach had to be used for the deep feature extraction. The lack of an external validation set means our results cannot be used for predictive inference; the necessity for cross-validation may have yielded overly optimistic results which, however, do not influence the comparison of the radiomic and deep features, which was the main objective of our investigation.

## 5. Conclusions

In our study, we compared the performance of deep features and radiomic features in classifying AIP or PDAC in portal venous CT-scans. We found that deep features achieved the best performance in discriminating both entities. Feature map visualization shows visually detectable difference, which can further improve radiological decision-making. The benefit of CNNs and deep features trained on CT-images of large AIP and PDAC cohorts should be investigated in following studies.

## Figures and Tables

**Table 1:** Clinical and histological parameters for autoimmune pancreatitis (AIP) and pancreatic ductal adenocarcinoma (PDAC).

Variable	AIP (*n* = 44)	PDAC (*n* = 42)
Age (Years)	Mean: 57 ± 17.3	Mean:67 ± 10.6
	Range: 26–82	Range: 34–88
Sex	Male: 29 (66%)	Male: 19 (45%)
	Female: 15 (34%)	Female: 23 (55%)
Focal/Multifocal/Diffuse	Focal: 30 (68%)	
	Multifocal: 2 (5%)	
	Diffuse: 12 (27%)	
Localisation (focal)	Head: 13 (43%)	Head: 30 (71%)
	Body: 4 (14%)	Body: 9 (21%)
	Tail: 13 (43%)	Tail: 3 (8%)

## Supplementary Materials

The following are available online at https://www.mdpi.com/2077-0383/9/12/4013/s1, Feature extraction and hyperparameter tuning; Figure S1: Flowchart showing patient recruitment of AIP and PDAC patients; Figure S2: Feature map visualization; Table S1: Exclusion criteria, Table S2: STROBE checklist.
