# Supplementary material for: Deep Convolutional Neural Network-Assisted Feature Extraction for Diagnostic Discrimination and Feature Visualization in Pancreatic Ductal Adenocarcinoma (PDAC) versus Autoimmune Pancreatitis (AIP)
Source: J Clin Med. 2020 Dec 11;9(12):4013. doi: 10.3390/jcm9124013 (PMC7764649; doi:10.3390/jcm9124013)
Supplement: Supplementary file 1 [file jcm-09-04013-s001.pdf]

Deep convolutional neural network-assisted feature extraction for diagnostic discrimination and feature visualization in pancreatic ductal adenocarcinoma (PDAC) versus autoimmune pancreatitis (AIP)

Supplementary material:

Feature extraction and hyperparameter tuning:

PyRadiomics version 3.0 was used for the analysis. Intensity discretization was performed to a fixed bin number of 25 bins. Feature normalization to the (0,1) interval was performed. Images were spatially resampled to 1x1x1mm using the BSpline interpolator. All first order statistics, shape-based, Gray Level Run Length Matrix, Gray Level Size Zone Matrix, Neighbouring Gray Tone Difference Matrix and Gray Level Dependence Matrix features and all Gray Level Cooccurence Matrix features except SumAverage (due to redundancy), as well as Laplacian of Gaussian-filtered (with Sigma value 1.0), wavelet-decomposition-based (using the coiflet 1 function), square, exponential, gradient, square-root and logarithm filtered versions of these features. GLCM and GLRLM were extracted using the default settings (separately for each direction then averaged). Feature descriptions can be found in the PyRadiomics documentation. 1411 features were extracted in total. The following hyperparameters were retained: *n* estimators = 200,max_depth = 15, max_features="sqrt", min_samples_leaf = 2 and min_samples_split = 2.

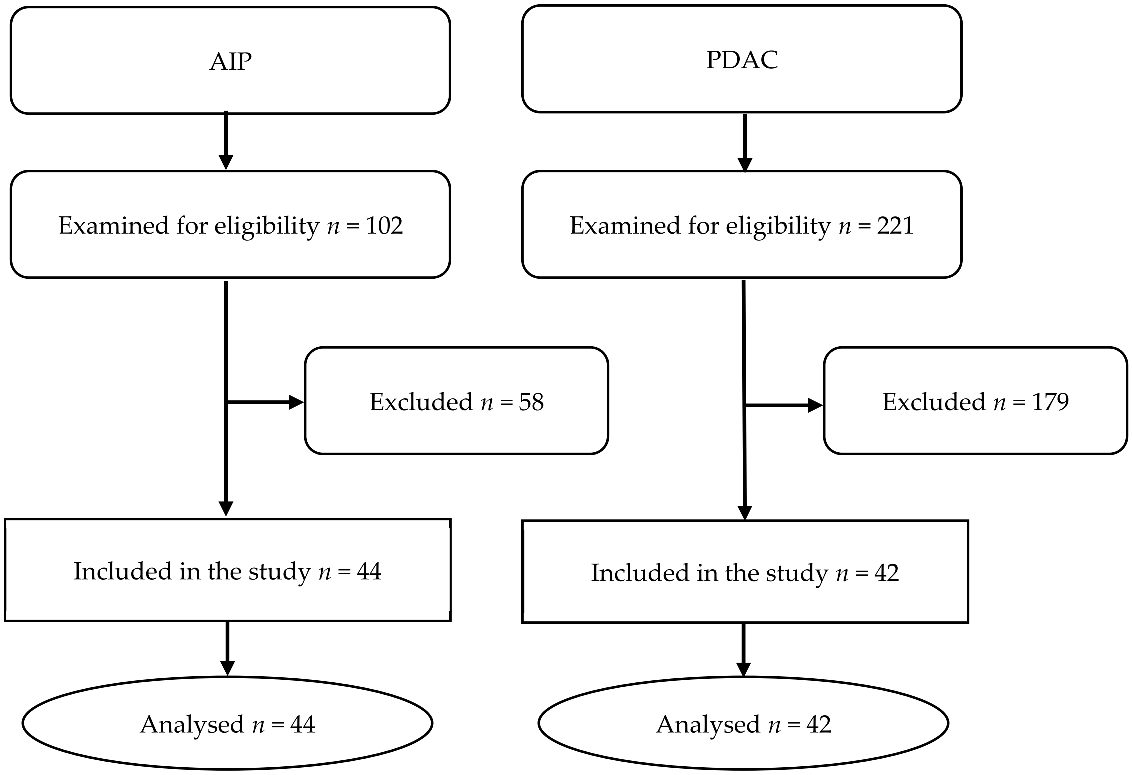

Figure S1. Flowchart showing patient recruitment of AIP and PDAC patients.

A

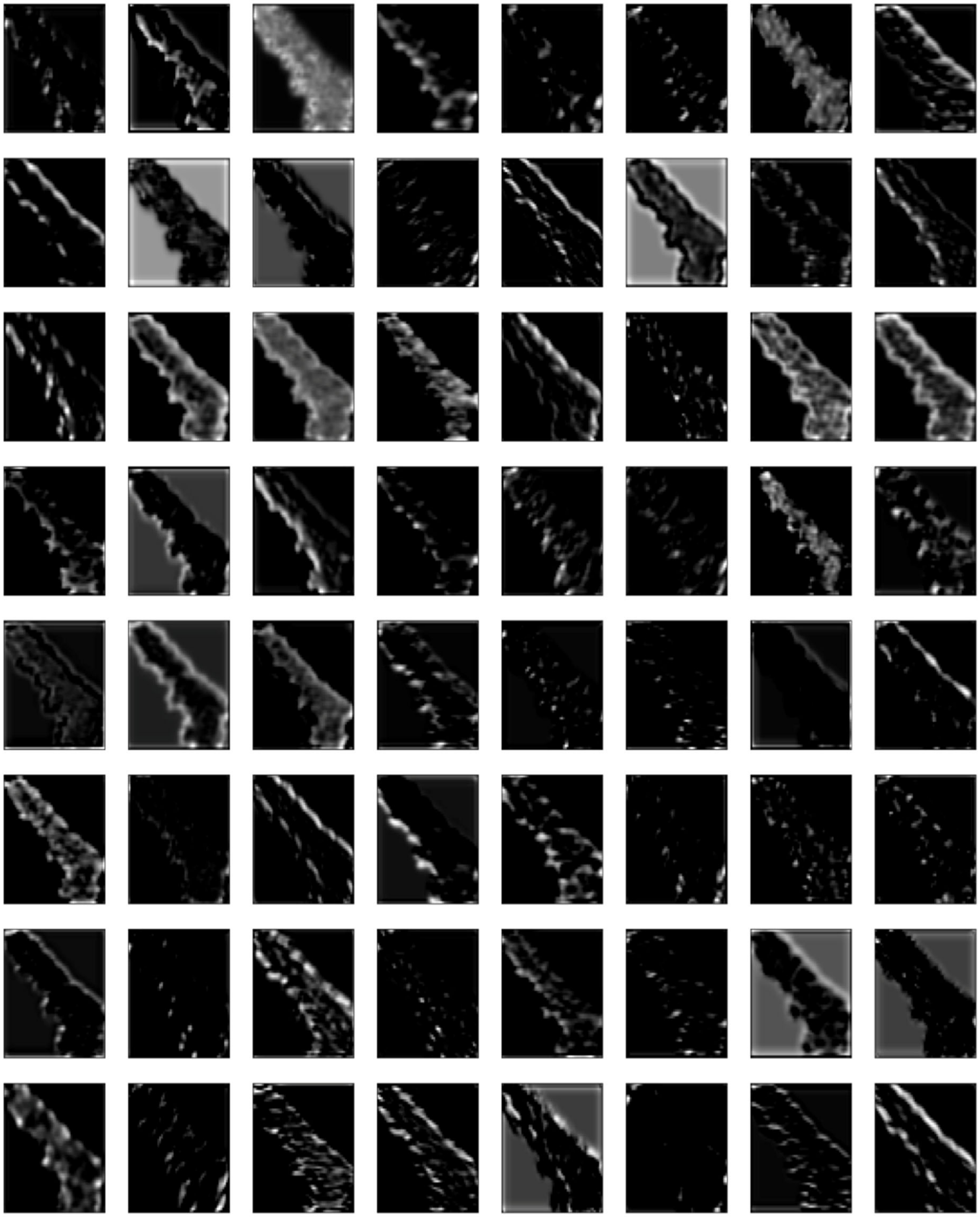

B

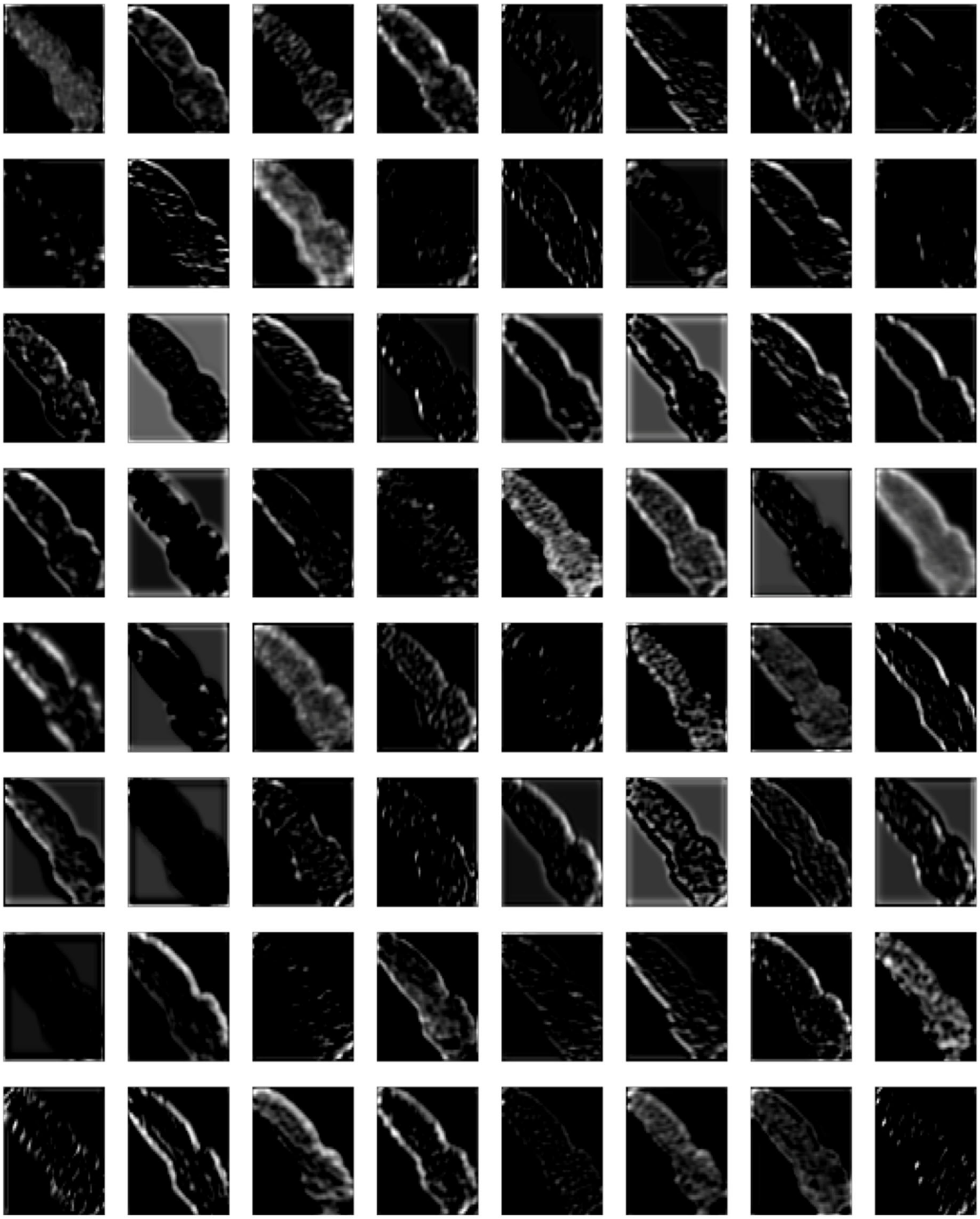

Figure S2. Feature map visualization**.** (A) Visualization of a subset (*n* = 64) of feature maps of the PDAC test patient. (B) Visualization of a subset (*n* = 64) of feature maps of the AIP test patient.

Table S1. Exclusion criteria.

**Reasons for exclusion (AIP):**	**Reasons for exclusion (PDAC):**
No computed tomography available (*n* = 24)	No computed tomography available (*n* = 33)
Insufficient contrast in portal venous phase (*n* = 13)	Insufficient contrast in portal venous phase (*n* = 25)

AIP eventually excluded (*n* = 21)	Tumorstage greater than T2 or enlarged lymph nodes in computer tomography according to the *Response Evaluation Criteria In Solid Tumors* (RECIST 1.1)(*n* = 121)

Table S2. STROBE checklist.

	Item No	Reccomendation	Remark/ Location
Title and abstract	1	(a) Indicate the study's design with a commonly used term in the title or the abstract	Abstract
		(b) Provide in the abstract an informative and balanced summary of what was done and what was found	Abstract
		Introduction	
Background/rationale	2	Explain the scientific background and rationale for the investigation being reported	Abstract, Introduction
Objectives	3	State specific objectives, including any prespecified hypotheses	Abstract Introduction, Discussion
		Methods	
Study design	4	Present key elements of study design early in the paper	Methods
Setting	5	Describe the setting, locations, and relevant dates, including periods of recruitment, exposure, follow-up, and data collection	Ibid.
Participants	6	(a) Give the eligibility criteria, and the sources and methods of selection of participants. Describe methods of follow-up	Methods, Supplementary material
		(b) For matched studies, give matching criteria and number of exposed and unexposed	Not applicable
Variables	7	Clearly define all outcomes, exposures, predictors, potential confounders, and effect modifiers. Give diagnostic criteria, if applicable	Methods, Results
Data sources/ measurement	8	For each variable of interest, give sources of data and details of methods of assessment (measurement). Describe comparability of assessment methods if there is more than one group	Methods
Bias	9	Describe any efforts to address potential sources of bias	Results, Discussion
Study size	10	Explain how the study size was arrived at	Methods, Supplementary material
Quantitative variables	11	Explain how quantitative variables were handled in the analyses. If applicable, describe which groupings were chosen and why	Methods, Table 1
Statistical methods	12	(a) Describe all statistical methods, including those used to control for confounding	Methods
		(b) Describe any methods used to examine subgroups and interactions	Ibid.
		(c) Explain how missing data were addressed	Methods, Discussion
		(d) If applicable, explain how loss to follow-up was addressed	Not applicable
		(e) Describe any sensitivity analyses	Results,

			Discussion
		Results	
Participants	13	(a) Report numbers of individuals at each stage of study—eg numbers potentially eligible, examined for eligibility, confirmed eligible, included in the study, completing follow-up, and analysed	Methods, Supplementary Material
		(b) Give reasons for non-participation at each stage	Ibid.
		(c) Consider use of a flow diagram	Supplementary material
Descriptive data	14	(a) Give characteristics of study participants (eg demographic, clinical, social) and information on exposures and potential confounders	Methods, Table 1,
		(b) Indicate number of participants with missing data for each variable of interest	Methods
		(c) Summarise follow-up time (eg, average and total amount)	Not applicable
Outcome data	15	Report numbers of outcome events or summary measures over time	Methods, Results
Main results	16	(a) Give unadjusted estimates and, if applicable, confounder-adjusted estimates and their precision (eg, 95% confidence interval). Make clear which confounders were adjusted for and why they were included	Results
		(b) Report category boundaries when continuous variables were categorized	Not applicable
		(c) If relevant, consider translating estimates of relative risk into absolute risk for a meaningful time period	Not applicable
Other analyses	17	Report other analyses done—eg analyses of subgroups and interactions, and sensitivity analyses	Results
		Discussion	
Key results	18	Summarise key results with reference to study objectives	Discussion
Limitations	19	Discuss limitations of the study, taking into account sources of potential bias or imprecision. Discuss both direction and magnitude of any potential bias	Discussion
Interpretation	20	Give a cautious overall interpretation of results considering objectives, limitations, multiplicity of analyses, results from similar studies, and other relevant evidence	Discussion
Generalisability	21	Discuss the generalisability (external validity) of the study results	Discussion
		Other information	
Funding	22	Give the source of funding and the role of the funders for the present study and, if applicable, for the original study on which the present article is based	Preamble
